# Visual Modulation of Acoustic Reflex Measurements: Insights Into Sensory Integration

**DOI:** 10.1002/brb3.70574

**Published:** 2025-05-18

**Authors:** Aysun Parlak Kocabay, Aysegul Esdogan, Aysenur Aykul Yagcioglu

**Affiliations:** ^1^ Department of Audiology Hacettepe University Ankara Turkey; ^2^ Department of Audiology KTO Karatay University Konya Turkey

**Keywords:** acoustic reflex latency, acoustic reflex threshold, cross‐modal interaction, sensory integration, visual task

## Abstract

**Objective:**

The aim was to investigate the impact of visual tasks on acoustic reflex thresholds (ARTs) and AR latencies (ARLs) during AR measurements.

**Methods:**

A total of 31 participants (17 female, 14 male; mean age: 20.3 ± 1.8 years) with normal hearing and Type A tympanograms were included. ARTs and latencies were measured using a 226 Hz probe tone under five distinct conditions: (1) resting with no visual task, (2) eyes comfortably closed, (3) fixed gaze on a stationary object, (4) saccadic eye movements, and (5) optokinetic eye movements. Data were analyzed using repeated measures ANOVA with Bonferroni correction applied for post hoc comparisons.

**Results:**

ARTs were significantly lower in the condition of eyes closed compared to fixed gaze, at both 1 and 2 kHz (*p* < 0.05). ARLs at 500 Hz were significantly shorter in the condition of fixed gaze compared to saccadic eye movements (*p* < 0.05). A significant interaction between frequency and condition was observed for ARTs, indicating frequency‐specific modulation by visual tasks, whereas no significant frequency and condition interaction was found for ARLs.

**Conclusion:**

Visual tasks have a measurable effect on ARTs and latencies, likely due to cross‐modal interactions between the auditory and visual systems. These results highlight the importance of standardizing visual and attentional conditions during clinical AR testing. Further research is warranted to elucidate the underlying neural mechanisms and their clinical implications.

## Graphical abstract

Visual tasks have a measurable effect on acoustic reflex thresholds and latencies, likely due to cross‐modal interactions between the auditory and visual systems.

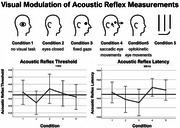

## 1 Introduction

The acoustic reflex (AR) is a protective mechanism that reduces the intensity of sound by contracting the stapedius muscle located in the middle ear in response to high‐intensity acoustic stimuli (Prabhu et al. 2015). A high‐intensity auditory stimulus triggers the contraction of the stapedius muscle, leading to the stiffening of the ossicular chain in both ears and a consequent decrease in energy transmission to the inner ear. The responses are reflexive, occurring naturally without necessitating an active response or overt emotion from the listener (Prendergast et al. 2024). AR testing is commonly utilized in clinical audiology to assess the integrity of the auditory system (Tasko et al. 2022).

Clinical AR measurement is typically performed using a 226 Hz probe tone in conjunction with a reflex‐activating stimulus presented to either the ipsilateral or contralateral ear. Measurements are generally conducted at the tympanometric peak pressure obtained through tympanometry, using either pure tones (500, 1000, 2000, or 4000 Hz) or broadband noise. An AR is considered present if the magnitude of the AR amplitude exceeds 0.02 mmho (Schairer et al. 2013). The AR threshold (ART) is defined as the minimum intensity level of an acoustic stimulus at which a minimal change in middle ear compliance can be detected. Literature indicates that the auditory response threshold in individuals with normal hearing generally ranges from 70 to 100 dB HL. AR latency (ARL) refers to the time interval between the onset of a high‐intensity acoustic stimulus and the initiation of stapedius muscle contraction. At stimulus levels just above the reflex threshold, latencies range from 150 to 250 ms. As the stimulus intensity increases, latency decreases. The shortest latency in AR is approximately 25 ms, recorded at stimulus levels 30–40 dB above the reflex threshold (Narayanan 2017). For normal AR measurement, the following conditions must be met: normal middle ear function, intact auditory nerve function, functional brainstem pathways between the cochlear nuclei and the superior olivary complex, and a normal facial nerve, including its stapedial branch. Additionally, the individual's hearing threshold should not exceed 60 dB HL. If one or more of these conditions are not satisfied, characteristic abnormalities in the reflex, such as prolonged latencies or reduced amplitudes, may be observed (Narayanan 2017).

The clinical applications of AR measurement include estimating hearing thresholds, differential diagnosis of conductive, cochlear, and retrocochlear pathologies, assessing facial nerve dysfunction, and detecting functional hearing loss (Mazlan et al. 2009). Various secondary tasks performed during AR measurement have been reported to influence the outcomes of the measurement (Corcoran et al. 1980; Prendergast et al. 2024; Robinette and Snyder 1982). However, to the best of the authors’ knowledge, no studies have been identified that specifically investigate the effects of secondary tasks on ARL. The aim of this study is to examine the impact of various visual tasks performed during AR measurement on ART and ARL findings. This study represents a novel approach to understanding the interaction between visual tasks and AR measurements, emphasizing the cross‐modal influence of visual and auditory systems. By examining the effects of various visual conditions on ARTs and latencies, this research provides critical insights into the underlying mechanisms of sensory integration and its clinical implications. These findings not only contribute to the growing body of knowledge regarding the modulation of auditory responses by non‐auditory stimuli but also underline the importance of controlling external variables, such as attention and visual tasks, during audiological assessments. The outcomes of this study could lead to the refinement of diagnostic protocols and enhance the accuracy of clinical evaluations involving AR testing.

## 2 Materials and Methods

The participants were informed about the objectives and scope of the study with the written consent form. All procedures were in accordance with the Helsinki Declaration (JAMA 2000; 284:3043–3049). The study was approved by the Local Ethical Committee (2024/047).

The study included 31 individuals (17 female, 14 male) aged 18–25 years (20.3 ± 1.8) with normal hearing, no ear pathology, and a Type A tympanogram according to the Jerger classification in tympanometry measurements.

### 2.1 Pure Tone Audiometry

Pure tone audiometry testing was conducted using the Interacoustics AC40 clinical audiometer and TDH39 supra‐aural headphones. The participants’ hearing thresholds were evaluated for octave frequencies ranging from 250 to 4000 Hz. Individuals with thresholds of 20 dB HL or better at these frequencies in both ears were considered to have normal hearing.

### 2.2 Tympanometry Measurement

Tympanometry testing was performed on individuals with normal hearing using the Interacoustics TITAN tympanometer. The tympanometry measurement was conducted using a 226 Hz probe tone.

### 2.3 AR Measurement

AR measurements were conducted on all subjects with the Interacoustics TITAN equipment under five distinct conditions. Reflex thresholds and latencies were assessed at frequencies of 500, 1000, and 2000 Hz using a 226 Hz probe tone for each condition. All measurements were performed on the right ear, and a compliance reduction of at least 0.02 mmho was considered a reflex response.

AR measurements were performed for each participant under the following five conditions:

Condition 1 The participant was instructed to remain still and not produce any sound while routine AR measurements were taken.

Condition 2 The participant was asked to close their eyes comfortably without squeezing, and AR measurements were conducted.

Condition 3 The participant was instructed to fixate on a stationary object located 1 m away, at a 0‐degree azimuth, and at eye level, without moving their eyes while AR measurements were taken.

Condition 4 AR measurements were conducted while the participant performed saccadic eye movements.

Condition 5 AR measurements were taken while the participant performed optokinetic eye movements.

The five test conditions were presented in a pseudo‐random order for each participant to avoid order effects. The VisualEyes 4 Channel Spectrum 9.1 videonystagmography device (Micromedical Technologies Inc., USA) and goggles were used for the fourth and fifth conditions of the AR measurement. A full HD LED television (Philips 5500 series, model 43PFS5505/62, 43‐in.) was used for object tracking. Before the tests, participants were seated 1 m away from the screen, and they were instructed to follow a green dot on the screen with their eyes only, without moving their heads, to calibrate the horizontal and vertical axes of the goggles.

Following calibration, the saccade test was conducted for the fourth condition, and the optokinetic test was used for the fifth condition. During the saccade test, participants were instructed to focus on randomly moving dots presented on the screen in the horizontal plane, which appeared at intervals of 2–3 s for a total of 30 movements. They were instructed to maintain their gaze fixed on the dot as long as it remained stationary in a specific position. For the optokinetic test, participants were instructed to follow dots moving horizontally across the screen at a speed of 30°/s, moving from left to right and right to left along the center of the screen.

Throughout these conditions, AR measurements were performed simultaneously with the specified tasks. ART and ARL data were collected for each condition.

### 2.4 Statistical Analysis

The data analysis was conducted using SPSS 25 (IBM Corp. released 2017. IBM SPSS Statistics for Windows, Version 25.0. Armonk, NY: IBM Corp.). Homogeneity of variances was assessed using the Levene test, whereas the normality assumption was evaluated using the Shapiro–Wilk test. ARTs and latencies were analyzed across five different conditions. To determine changes in thresholds and latencies across measurements based on the number of categorical variables, repeated measures analysis of variance (ANOVA) was used. When significant differences were identified between groups, Bonferroni correction was applied to pinpoint these differences. The assumption of sphericity was assessed using Mauchly's test. When the sphericity assumption was met, the sphericity assumed test was applied. If the assumption was violated, the epsilon value was examined: The Huynh–Feldt test was used when the epsilon value exceeded 0.75, whereas the Greenhouse–Geisser test was applied when the epsilon value was below 0.75. A *p* value of <0.05 was considered statistically significant.

## 3 Results

The repeated measures ANOVA was conducted with condition (C1–C5) and stimulus frequency (500, 1000, 2000 Hz) as within‐subject factors. The main effect of frequency on ARTs was not significant (*F*(2,60) = 1.213, *p* = 0.305). However, the main effect of condition was significant (*F*(4,120) = 3.054, *p* = 0.019, *η* ^2^ = 0.092), and a significant interaction between frequency and condition was also found (*F*(8,240) = 2.45, *p* = 0.021). Further analyses by frequency revealed that for 500 Hz, the condition effect was not significant (*F*(4,120) = 1.552, *p* = 0.192, *η* ^2^ = 0.049). For 1000 Hz, the condition effect was significant (*F*(4,120) = 3.214, *p* = 0.015, *η* ^2^ = 0.097), and for 2000 Hz, it was also significant (*F*(4,120) = 3.054, *p* = 0.019, *η* ^2^ = 0.092).

Bonferroni‐corrected post hoc comparisons indicated that at 1 and 2 kHz, thresholds were significantly lower in Condition 2 (eyes closed) compared to Condition 3 (fixed gaze) (*p* < 0.05). Table 1 presents the mean, *F*, and *p* values of the ARTs obtained under different conditions.

**TABLE 1:** Acoustic reflex thresholds in different conditions.

Frequency (kHz)	Condition	Mean	*F*	*p*
**0.5**	C1	84.03 ± 4.73	1.55	0.20
	C2	83.55 ± 4.86		
	C3	84.35 ± 5.28		
	C4	84.84 ± 5.08		
	C5	84.52 ± 5.06		
**1**	C1	83.87 ± 4.6	3.21	0.03*
	C2*	82.42 ± 4.45		
	C3*	84.35 ± 5.59		
	C4	83.71 ± 4.82		
	C5	83.23 ± 4.75		
**2**	C1	85.65 ± 5.59	3.05	0.03*
	C2*	84.35 ± 4.79		
	C3*	86.77 ± 5.85		
	C4	85.97 ± 5.97		
	C5	85.48 ± 5.38		

*: indicates a statistically significant difference at *p*<.05

**FIGURE 1:**
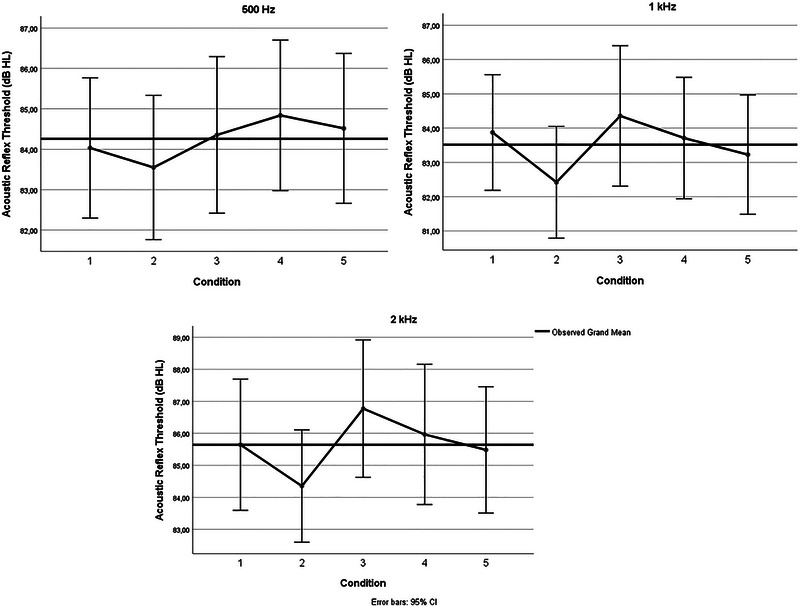
Acoustic reflex thresholds under different conditions on 0.5, 1, and 2 kHz.

**FIGURE 2:**
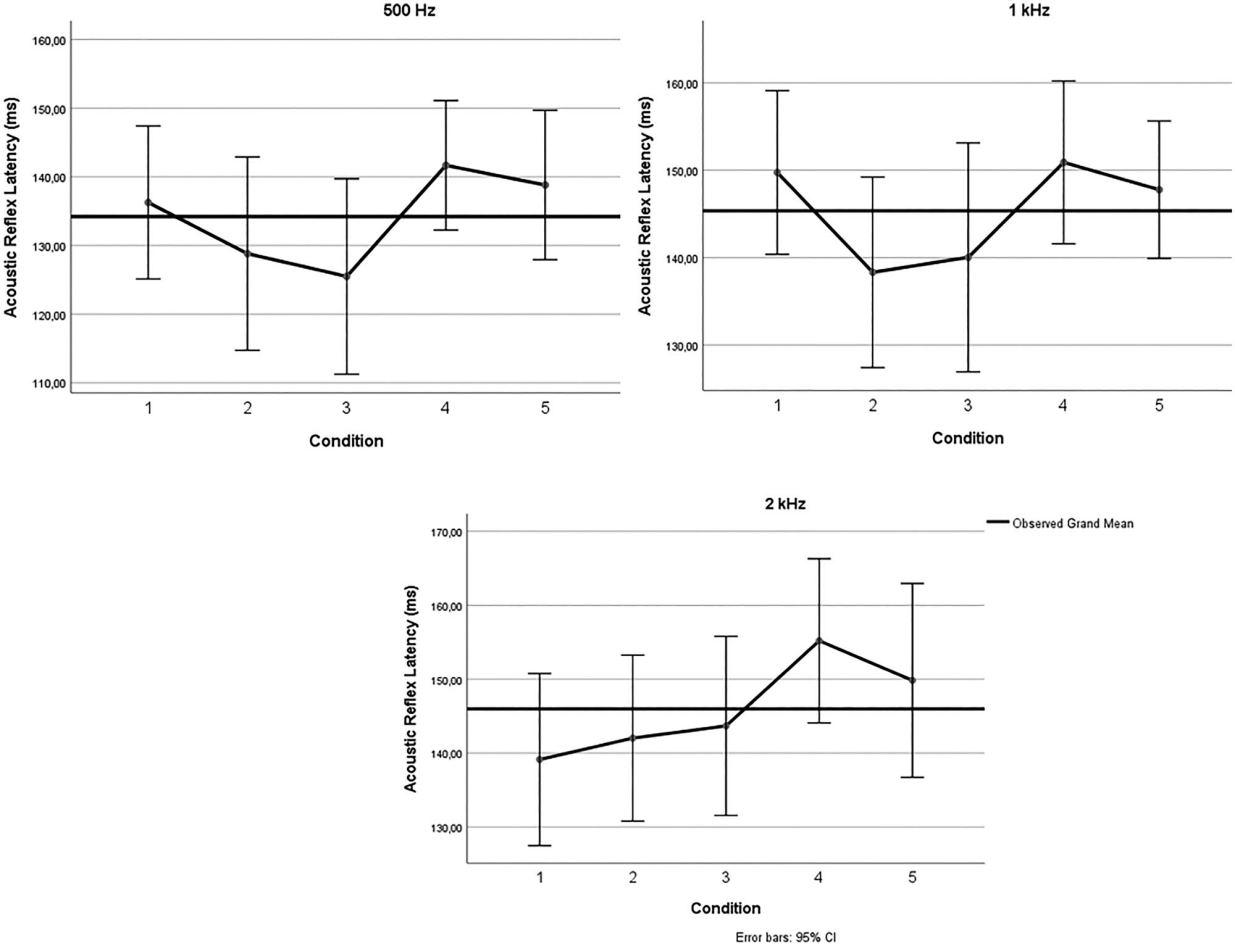
Acoustic reflex latencies under different conditions on 0.5, 1, and 2 kHz.

For ARLs, the repeated measures ANOVA showed a significant main effect of condition (*F*(4,120) = 3.054, *p* = 0.019, *η* ^2^ = 0.092), but no significant main effect of frequency (*F*(2,60) = 1.213, *p* = 0.305), and no significant interaction between frequency and condition (*F*(8,240) = 1.572, *p* = 0.135). Further analyses by frequency revealed that for 500 Hz, Bonferroni‐corrected post hoc comparisons showed that latencies were significantly shorter in Condition 3 (fixed gaze) compared to Condition 4 (saccadic eye movements) (*p* < 05). For 1000 and 2000 Hz, no significant condition differences were found. The mean, *F*, and *p* values are presented in Table 2. Figures 1 and 2 show the ARTs and ARLs under different conditions, respectively.

**TABLE 2:** Acoustic reflex latancies in different conditions.

Frequency (kHz)	Condition	Mean	*F*	*p*
**0.5**	C1	136.3 ± 30.4	3.21	0.03*
	C2	128.8 ± 38.4		
	C3*	125.5 ± 38.8		
	C4*	141.7 ± 25.7		
	C5	138.8 ± 29.7		
**1**	C1	149.7 ± 25.6	2.36	0.09
	C2	138.3 ± 29.7		
	C3	140.1 ± 35.7		
	C4	150.9 ± 25.4		
	C5	147.8 ± 21.4		
**2**	C1	139.1 ± 31.8	2.12	0.11
	C2	142.1 ± 30.6		
	C3	143.7 ± 33.1		
	C4	155.2 ± 30.3		
	C5	149.8 ± 35.8		

*: indicates a statistically significant difference at *p*<.05

## 4 Discussion

This study investigates the impact of visual tasks on ARTs and ARL, proposing the hypothesis that visual tasks may modulate middle ear muscles, influencing the neural processing of auditory pathways. The results of this study highlight the significant influence of visual tasks on ART and ARL, demonstrating a clear interaction between the auditory and visual systems. These findings align with the previous research indicating that sensory systems are not isolated but interact dynamically to modulate neural responses. The lower ARTs observed during the eyes‐closed condition suggest that middle ear muscle activity may be enhanced by concurrent eye muscle activity, supporting hypotheses of cross‐modal integration. Additionally, the prolonged ARLs during saccadic eye movements reveal the potential impact of attention and visual task complexity on auditory reflex pathways. These findings provide new perspectives on the functional interplay between sensory modalities, with implications for both basic research and clinical practice.

Middle ear muscle contractions, although typically triggered in response to high‐level acoustic stimuli, can also be observed in response to somatosensory stimulation or in conjunction with other motor activities, even in the absence of any acoustic input. Tasko et al. (2022) investigated the relationship between middle ear muscle contractions and eyelid closure effort using EMG recordings of the orbicularis oculi muscle. Their study showed that middle ear muscle contractions occurred during eyelid closure movements tracked via EMG. The presence and magnitude of these contractions increased with higher levels of eyelid closure effort. These findings revealed a positive correlation between middle ear muscle activity and eye muscle activity (Tasko et al. 2022). The reduced ART values observed in Condition 2 (eyes closed) compared to Condition 3 (fixed gaze) may be explained by the enhanced activation of middle ear muscles via concurrent eye muscle activity, as suggested by Tasko et al. (2022). This hypothesis aligns with their findings that orbicularis oculi muscle contractions during eyelid closure could correlate with middle ear muscle activity. Further electrophysiological studies, potentially incorporating EMG recordings, could provide a more detailed understanding of this mechanism.

Attention is the capacity of cognitive structures to focus on particular inputs while disregarding others. It is thought that during states of attention, neuronal activity in the auditory centers of the brain undergoes alterations (Delano et al. 2007). Literature indicates that auditory or visually directed attention can modulate the auditory system (Gruters et al. 2018). Oatman (1976) reported a reduction in the amplitude of auditory nerve compound action potentials (CAP) during visual attention in cats (Oatman 1976). In a related study, Lukas (1980) demonstrated that participants engaged in counting flashing letters on a screen exhibited a significant 16.4% decrease in the amplitude of wave V generated from the inferior colliculus, accompanied by a latency increase of 27 µs (Lukas 1980). Similarly, Puel et al. (1988) reported a decline in the amplitude of evoked otoacoustic emission responses during tasks requiring selective attention (Puel et al. 1988). Delano et al. (2007) investigated the impact of selective attention on cochlear sensitivity by training chinchillas in a two‐alternative forced‐choice task involved visual and auditory frequency discrimination. Their findings revealed a significant reduction in cochlear sensitivity, as indicated by a decrease in CAP amplitude, during visual attention in animals performing the visual discrimination task. Conversely, this reduction was not observed in animals engaged in the auditory discrimination task. These results suggest that the observed physiological effects are attributable to selective attention directed toward visual stimuli rather than an overall increase in arousal levels (Delano et al. 2007). The findings of our study provide the statistically significant differences in ART and ARL under varying visual conditions that highlight the modulatory effects of visual attention and eye movement on auditory reflex pathways. The prolonged ARL observed in Condition 4 (saccadic eye movements) compared to Condition 3 (fixed gaze) at 500 Hz supports the notion that complex visual tasks requiring dynamic attention allocation can influence the temporal dynamics of the AR. This effect may stem from the neural competition for processing resources in the brainstem auditory pathways during visually demanding tasks. Such an explanation is consistent with Delano et al. (2007) and Gruters et al. (2018), who demonstrated that visual attention could modulate auditory system activity, leading to latency variations and reduced neural amplitudes (Delano et al. 2007; Gruters et al. 2018). Moreover, the presence of a significant condition and frequency interaction for ARTs indicates that visual modulation of ARs may vary depending on stimulus frequency, a novel contribution to cross‐modal research. This frequency‐specific modulation aligns with prior findings that attentional or cognitive demands can alter sensory thresholds in a load‐dependent manner (Mahjoob and Anderson 2023, 2024).

The broader implications of these findings suggest that the AR is not solely an auditory phenomenon but is also susceptible to cross‐modal influences. This observation is particularly relevant for clinical applications of AR testing, as it emphasizes the importance of standardizing experimental conditions to minimize the impact of extraneous variables. Future research should explore the underlying neural circuits connecting the visual and auditory pathways, with a focus on elucidating how these interactions vary across different sensory modalities and task complexities. Additionally, studies investigating the role of selective attention in modulating cochlear and middle ear function could further enhance our understanding of sensory integration in auditory diagnostics.

## 5 Conclusion

This study demonstrates that visual tasks performed during AR measurements can significantly influence both ARTs and latencies, depending on task complexity and stimulus frequency. The findings suggest that eye muscle activity and visual attention may modulate middle ear muscle function, potentially altering reflex responses. These results underscore the necessity of controlling visual and attentional variables during clinical AR testing to ensure consistent and reliable measurements. Moreover, the cross‐modal interactions between the visual and auditory systems observed in this study highlight the complexity of sensory integration within the central nervous system. Understanding these interactions may contribute to refining diagnostic protocols for auditory and vestibular pathologies. Future research should aim to further investigate the neural mechanisms underlying these effects and their clinical implications.

## Author Contributions

**Aysun Parlak Kocabay**: conceptualization, methodology, formal analysis, writing – original draft, writing – review and editing, project administration. **Aysegul Esdogan**: conceptualization, methodology, investigation, data curation, writing – original draft. **Aysenur Aykul Yagcioglu**: investigation, data curation, formal analysis, writing – original draft, visualization.

## Conflicts of Interest

The authors declare no conflicts of interest.

### Peer Review

The peer review history for this article is available at https://publons.com/publon/10.1002/brb3.70574.

## Data Availability

The data that support the findings of this study are available on request from the corresponding author. The data are not publicly available due to privacy or ethical restrictions.
